# Evaluation for Fatty Liver Infiltration Should Become Standard in Pediatric Cholecystectomy Patients

**DOI:** 10.7759/cureus.99219

**Published:** 2025-12-14

**Authors:** Mary M Barron, Lindsay H Devereux, Nathan Dockery, Marianne Neal, Trevy Ramos, Brad Feltis

**Affiliations:** 1 Surgery, East Tennessee State University, Johnson City, USA; 2 Center for Rural Health and Research, East Tennessee State University, Johnson City, USA; 3 Radiology, East Tennessee State University, Johnson City, USA; 4 Pediatric Surgery, East Tennessee State University, Johnson City, USA

**Keywords:** cholecystectomy, fatty liver disease, gallbladder disease, gallbladder removal, hepatic steatosis, masld, ultrasound screening

## Abstract

Introduction

Pediatric metabolic dysfunction-associated steatotic liver disease (MASLD) is rapidly becoming a health crisis. Despite clear benefits of recognition and treatment, optimal methods of detection have not been established. Children with known cholelithiasis may be at increased risk for fatty infiltration of their liver, presenting an opportunity for screening using ultrasound.

Methods

A retrospective chart review was conducted on children undergoing cholecystectomy at an institution in rural Appalachia over three years. A pediatric radiologist re-evaluated ultrasounds for fatty liver infiltration. Data were analyzed with respect to BMI. Clinical indicators associated with MASLD on the presence of fatty liver infiltration.

Results

Sixty-two patients met inclusion criteria; 10 had ultrasound evidence of fatty liver infiltration (16%) and they were more likely to have a higher median weight percentage (98.7 vs. 94.5, p=0.0080). Individuals who were obese or severely obese had a significantly higher blood alanine aminotransferase (ALT) than those who were not obese (43 vs 16, p=0.0432).

Conclusion

Our study provides evidence that children with obesity are at greater risk for developing fatty infiltration of the liver and potentially MASLD. Evaluating a patient’s ultrasound for liver pathology at the time of gallbladder evaluation is a simple intervention that should become standard.

## Introduction

A recent special article authored by dozens of national and international authorities calls for “immediate attention” to curb a pending “health crisis” concerning pediatric metabolic dysfunction-associated steatotic liver disease (MASLD), formerly known as nonalcoholic fatty liver disease (NAFLD) [1-3]. MASLD is liver steatosis, or fatty liver disease (FLD), that is secondary to metabolic dysfunction [2]. The presence of FLD is determined by liver imaging or biopsy showing at least 5% macrovesicular steatosis [2,3]. To diagnose MASLD, the individual must not have another cause of liver steatosis, and they must have at least one cardiometabolic dysfunction risk factor [2]. For pediatric patients, there are five cardiometabolic dysfunction risk factors defined based on parameters of body mass index (BMI), serum glucose, blood pressure, plasma triglycerides, and plasma high-density lipoprotein (HDL) [2,4]. 

The estimated prevalence of MASLD has been reported to be 5-10% among the general population and 26-53% among children with obesity [5-7]. Recognizing MASLD and managing the condition with diet and exercise alone has proven beneficial in mitigating serious health consequences for these patients [8,9]. Despite clear benefits of recognition and treatment, optimal methods for detecting the disease in children have not yet been established [2,10,11]. Liver biopsy is the gold standard for diagnosis of FLD, though ultrasound has been shown to be predictive and therefore may be useful and practical as a screening tool in some clinical scenarios [2,12]. Liver enzymes alone are insufficient measures of MASLD, as aspartate aminotransferase (AST) and alanine aminotransferase (ALT) can be normal in up to two-thirds of patients with MASLD [2,13-15]. 

In 2023, the American Academy of Pediatrics (AAP) published the first clinical practice guideline concerning management of pediatric patients over the age of two years who have obesity [16]. In this clinical practice guideline, obesity is recognized as a risk factor for MASLD. They concluded that children with obesity aged 10 or older should be evaluated for abnormal liver function. However, within this guideline, there is no current recommendation for ultrasound evaluation of the liver, which would be a more accurate data point than liver enzyme levels. 

We hypothesized that children with known gallbladder disease may be at increased risk for fatty infiltration of the liver and if found, should be referred for MASLD evaluation, as there can be interventions to reverse the steatotic process. Our primary goal of this novel, not previously presented retrospective review, was to determine if there is a relationship between weight class and prevalence of fatty liver infiltration in pediatric patients undergoing cholecystectomy. Secondarily, we aimed to determine if there is a relationship between weight class and surgical outcomes in our population. Previously, obesity has been determined to not be an independent risk factor for postoperative complications from cholecystectomies in children [17,18]. However, it has been linked to greater operative difficulty and prolonged operative times [17].

## Materials and methods

Measures

Cholelithiasis has a well-known standard definition of gallstones in the gallbladder, which encompasses more specific diagnoses such as calculous cholecystitis, choledocholithiasis, and gallstone pancreatitis [19]. Biliary dyskinesia is characterized by gallbladder dysfunction without evidence of mechanical obstruction [20]. The diagnosis is often supported by an abnormal ejection fraction measured by hepatobiliary iminodiacetic acid (HIDA) scan and can be further classified as biliary hypokinesia (ejection fraction <35-40%) or hyperkinesia (ejection fraction >80%) [20,21].

Data collection

After obtaining approval from the research institution’s IRB, a retrospective chart review was conducted on children aged one to 18 undergoing cholecystectomy at our institution in rural Appalachia between January 2020 and December 2022. Additional inclusion criteria are as follows: age between one to 18 years, documented diagnosis of cholelithiasis or biliary dyskinesia, a gallbladder ultrasound image available for review, and a laparoscopic cholecystectomy operative report. Exclusion criteria are as follows: any individual above the age of 18 or under the age of one year, no gallbladder ultrasound image available, or no cholecystectomy operative report. 

Collected variables include continuous measures of surgery duration (minutes), preoperative liver enzymes (AST and ALT, U/L) and total bilirubin (mg/dL), and categorical measures of preoperative diagnosis, surgical complications, ultrasound imaging results, and length of postoperative stay (days). Available ultrasounds were reviewed by a pediatric radiologist to determine the presence or absence of fatty liver infiltration. In order to simulate the clinical environment, the radiologist was not blinded to any patient information that they would normally have access to. All data were analyzed with respect to BMI percentile and category class based on taxonomy previously adopted by the CDC for children and teenagers: underweight, healthy weight, overweight, obese, severely obese (Table 1). Each patient’s BMI and category class were determined using the online CDC BMI Calculator for Child and Teen, which compares an individual’s BMI to others of the same sex and age.

**Table 1:** CDC BMI Categories for Children and Teens, Ages Two to 19 [22]

BMI Category	BMI Range
Underweight	Less than the 5th percentile
Healthy Weight	5th percentile to less than the 85th percentile
Overweight	85th percentile to less than the 95th percentile
Obesity	95th percentile or greater
Severe Obesity	120% of the 95th percentile or greater OR 35 kg/m2 or greater

Analysis

The analysis divided the population into two groups: “obese” compared to “not obese.” Based on the CDC categories, severely obese and obese patients were grouped together as “obese,” while overweight, healthy, and underweight patients were grouped together as “not obese” (Table 1). Characteristics of the sample population were described using percentages and frequencies or medians and interquartile ranges (IQR). Following the analyses, clinical indicators associated with fatty liver disease were analyzed based on the presence of fatty liver infiltration in the patient. Due to the non-normal distribution of the data, Mann-Whitney rank-sum tests were utilized for continuous variables. For categorical variables, Fisher’s exact tests were selected because of the small sample size of the data. All analyses were conducted in Stata version 18 (StataCorp., College Station, TX, USA) [23].

## Results

During the study period, 81 children underwent cholecystectomy, of whom 62 patients met the inclusion criteria; 46 had gallstone disease and 16 had biliary dyskinesia. Of those with biliary dyskinesia, 12 had hypokinesia and four had hyperkinesia. The median age of patients was 15 years old. Forty-eight patients were female, with the average length of surgery being 87 minutes. Twenty-four hospital stays were for less than 24 hours. Ten patients had ultrasound evidence of fatty liver infiltration (16%). Of those with fatty infiltration, eight were severely obese, one was overweight, and one was healthy. 

Individuals who were obese or severely obese had a significantly higher blood ALT than those who were not obese (43 vs 16, p=0.0432) (Table 2). Based on the laboratory test used, the upper limit of normal ALT is 41 and AST is 34. No significant differences were reported between the groups with respect to age, gender, length of surgery, length of stay, AST, bilirubin, or diagnosis (Table 2). 

**Table 2:** Descriptive statistics of data categorized as obese vs. not obese †Mann-Whitney U test conducted ‡Fischer’s exact test conducted *P ≤ 0.05 ALT: alanine aminotransferase, AST: aspartate aminotransferase

Variable	Total Sample (n=62) N (%) or median ± IQR	Severely Obese and Obese (n=34) N (%) or median ± IQR	Not Obese (n=28) N (%) or median ± IQR	P-value
Age (years)†	15 (13 - 16)	15 (14 - 16)	15 (12 - 16)	0.7040
Gender‡				0.7650
Male	14 (22.58%)	7 (20.59%)	7 (25.0%)	
Female	48 (77.42%)	27 (79.41%)	21 (75.0%)	
Length of Surgery (minutes)†	87 (75 - 106)	87 (76 - 107)	86 (65 - 101)	0.4243
Length of Stay‡				0.7190
Less than 24 hours	24 (38.71%)	12 (35.29%)	12 (42.86%)	
24 – 48 hours	34 (54.84%)	19 (55.88%)	15 (53.57%)	
Greater than 48 hours	4 (6.45%)	3 (8.82%)	1 (3.57%)	
ALT (U/L)†	34.5 (15 - 118)	43 (18 - 144)	16 (12 - 47)	0.0432*
AST (U/L)†	26 (17 - 63)	31 (19 - 99)	23 (14 - 48)	0.0742
Bilirubin (mg/dL)†	0.7 (0.3 - 1.2)	0.7 (0.4 - 1.3)	0.7 (0.3 - 1.1)	0.5213
Diagnosis‡				0.1470
Biliary Dyskinesia	16 (25.81%)	6 (17.65%)	10 (35.71%)	
Gallstone Disease	46 (74.19%)	28 (82.35%)	18 (64.29%)	

Data were also stratified according to whether an individual had ultrasound evidence of fatty infiltration. There was a significant difference between the weight percentages of patients who had fatty infiltration versus those who did not (Table 3). Individuals who had fatty infiltration of their liver were more likely to have a higher median weight percentage (98.7 vs. 94.5, p=0.0080) (Table 3). For length of surgery, liver enzymes, bilirubin levels, and diagnosis, no statistical significance was found according to the presence or absence of FLD. 

**Table 3:** Analysis of clinical factors and outcomes based on the presence of fatty liver disease †Mann-Whitney U test conducted ‡Fischer’s exact test conducted *P ≤ 0.05 ALT: alanine aminotransferase, AST: aspartate aminotransferase

Variable	Total Sample (N = 62)	Fatty Liver Present (N = 10)	Fatty Liver Not Present (N = 52)	P-Value
Weight Percentile†	95.2 (83 - 98.3)	98.7 (98.3 - 99.9)	94.5 (83 - 97.3)	0.0080*
Length of Surgery (minutes)†	87 (75 - 106)	87 (75 - 106)	86.5 (76 - 105)	0.9351
AST (U/L)†	26 (17 - 63)	34 (20 - 58)	25 (17 - 65)	0.6175
ALT (U/L)†	34.5 (15 - 118)	36 (18 - 108)	33 (13 - 118)	0.6176
Bilirubin (mg/dL)†	0.7 (0.3 - 1.2)	0.6 (0.5 - 0.7)	0.8 (0.3 - 1.3)	0.4770
Diagnosis‡				1.00
Biliary Dyskinesia	16 (25.81%)	2 (12.5%)	14 (87.5%)	
Gallstone Disease	46 (74.19%)	8 (17.4%)	38 (82.6%)	

## Discussion

Obesity and fatty liver infiltration 

Our study provides more evidence that children with obesity are at a greater risk for developing fatty infiltration of the liver and potentially MASLD. Because ultrasound has been shown to be useful as a screening tool for fatty infiltration [12], we hypothesized that fatty infiltration would be related to obesity in our study population. Therefore, each patient’s ultrasound was reviewed by a single pediatric radiologist specifically looking for fatty infiltration of the liver. On the initial read, not all patients had comments on presence or absence of fatty infiltration. This is not surprising, as typically a request for ultrasound of the gallbladder does not include inquiries about the condition of the surrounding liver parenchyma. Regarding the feasibility of routinely evaluating for MASLD on right upper quadrant ultrasounds obtained for gallbladder evaluation, expert pediatric radiologist opinion is that it does not require significant additional amount of time. Evaluation of the liver for echotexture and mass is part of every complete abdominal or right upper quadrant ultrasound. Evaluation for MASLD is included as part of this and is routinely done with no additional Imaging. 

In our study population, 42% of severely obese children were found to have fatty infiltration on retrospective review of their ultrasound. We propose that when providers order a gallbladder ultrasound, they should also ask the radiologist to examine the liver for presence or absence of hepatic steatosis. In these instances, if there is evidence of hepatic steatosis, children should be referred to a pediatric gastroenterologist for MASLD assessment. To diagnose MASLD, the individual must not have another cause of liver steatosis, and they must have at least one cardiometabolic dysfunction risk factor. 

Our study also found that there is a significant difference in ALT (p=0.04) and a trend towards significance in AST (p=0.07) based on whether the child is obese (Table 2). Interestingly, these differences were not present when comparing patients with or without fatty infiltration. Our data implies that, within the population of pediatric patients undergoing cholecystectomy, liver enzymes are not reliably elevated in the presence of steatosis, though elevated ALT may be associated with higher weight class. Others have also documented that serum transaminases are an unreliable indicator of fatty liver infiltration [13-15]. However, based on AAP recommendations, it may be appropriate to check the child’s ALT on an outpatient basis after their gallbladder pathology has resolved [16,24]. 

Early recognition and intervention in these children could prevent devastating consequences for their health. Although novel screening methods for MASLD in pediatric patients are being studied and developed [25-27], checking a patient’s ultrasound for liver pathology while they are already being examined for gallbladder pathology is a simple intervention that should become standard. Additionally, when a radiologist finds evidence of MASLD, the patient’s primary provider in the hospital should ensure that the child is connected with appropriate outpatient care for evaluation of their liver alongside a pediatric obesity medicine specialist.

BMI and surgical outcomes 

In this study, weight class was not a predictor of biliary pathology, surgical complications, increased operative time, or length of stay for children undergoing laparoscopic cholecystectomy (Table 2). Previously, some studies have documented increased operative time associated with obesity in this population [17]. We speculate that the average years in practice of each pediatric surgeon (30) contributed to our efficiency with this operation. 

Although the literature is sparse, previous investigators have also not found differences in surgical outcomes for pediatric cholecystectomy patients based on whether they are obese [17,18]. Our lack of significant differences in operative outcomes based on weight is therefore not surprising. Of note, we also conducted an analysis that divided the population based on whether they were overweight: severely obese, obese, and overweight compared to healthy and underweight. We found no significant difference between this analysis and the one we present.

Limitations

Despite our findings, this study is not without limitations. This is a cross-sectional study that is concerned with only a singular span of time. As a result, causality cannot be determined. Our sample size and subsequent analytical dataset are small, which limits the power of the statistical analyses chosen. 

Concerning the generalizability of our findings, this study evaluates a population of children at a single children’s hospital in rural Tennessee. Although childhood obesity is more prevalent in Tennessee compared to the national average, this difference is relatively small [28]. Therefore, we argue that our study is likely generalizable across a broader distribution of children nationally. Ideally, we would have employed a control group consisting of children with normal HIDA scans and right upper quadrant ultrasounds revealing no gallbladder pathology. We are hopeful that future studies will employ such a group to expand upon the findings we present. 

Our study was unable to consider certain demographic information which may impact obesity rates and access to healthcare, such as race and socioeconomic status. We are hopeful to collect and analyze these factors in a follow-up study. Our study was unable to consider other confounding or risk factors including the presence of other medical conditions or medications that could affect liver health or obesity. This may lead to bias or masking of other significant factors. 

We recognize our study’s limitations, and we encourage future studies to consider these factors, as outlined above, in their research design and analysis.

## Conclusions

Children with obesity are at a greater risk for developing fatty infiltration of the liver and potentially MASLD. In our study, almost half of severely obese children (42%) were found to have fatty infiltration of their liver by ultrasound evaluation. Liver enzymes are not reliably elevated in the presence of steatosis, but ultrasound has been shown to be useful as a screening tool. Right upper quadrant ultrasounds obtained for gallbladder evaluation in pediatric patients include capturing images of liver parenchyma, but it is often not commented on. According to expert pediatric radiologist opinion, evaluating for MASLD on these ultrasounds does not require a significant additional amount of time. We propose that when providers order a gallbladder ultrasound, they should also ask the radiologist to examine the liver for presence or absence of hepatic steatosis. In these instances, if there is evidence of hepatic steatosis, children should be referred to a pediatric gastroenterologist for complete MASLD assessment. 

Early recognition and intervention in these children could prevent devastating consequences for their health. Although novel screening methods for MASLD in pediatric patients are being studied and developed, checking a patient’s ultrasound for liver pathology while they are already being examined for gallbladder pathology is a simple intervention that should become standard. Additionally, when a radiologist finds evidence of MASLD, the patient’s primary provider in the hospital should ensure that the child is connected with appropriate outpatient care for evaluation of their liver alongside a pediatric obesity medicine specialist.
